# Emotion-Focused Cognitive Behavioral Therapy in Comorbid Obesity With Binge Eating Disorder: A Pilot Study of Feasibility and Long-Term Outcomes

**DOI:** 10.3389/fpsyg.2020.00343

**Published:** 2020-03-05

**Authors:** Sandra Torres, Célia M. D. Sales, Marina Prista Guerra, Maria P. Simões, Mariana Pinto, Filipa M. Vieira

**Affiliations:** ^1^Faculty of Psychology and Education Sciences, University of Porto, Porto, Portugal; ^2^Center for Psychology, University of Porto (CPUP), Porto, Portugal

**Keywords:** obesity, binge eating disorder, emotional processing, emotional eating, cognitive behavioral therapy, group therapy

## Abstract

Obesity coupled with binge eating disorder (BED) is an increasing problem. Incorporation of treatment strategies that address both problems in people with comorbid symptoms is of major interest. This study aimed to investigate the feasibility and preliminary long-term outcomes of a brief emotion-focused cognitive behavioral therapy (EF-CBT) program. Seven out of 10 women with obesity and BED completed the intervention. Standardized outcome measures to assess the intensity of distress caused by complaints, psychological distress, emotional processing, eating behavior, and weight loss were completed at baseline, end-of-treatment, 6- and 18-month follow-ups. Individualized outcome measures were also applied to describe the personal experiences during the intervention. Findings suggest the program’s long-term efficacy for improving psychological distress, emotional processing, and alexithymia. Positive reliable changes in emotional processing and alexithymia were observed in almost all participants. The mean intensity of distress caused by complaints also decreased at the end of the intervention, with a large effect size (*d* = 0.89). Reliable changes in these outcome measures were observed at all time-points, despite the mean scores for the 18-month follow-up suggest a retraction in improvement. Weight loss was below expectations at all time-points, as were changes in emotional and external eating. Restrained eating mean scores remained stable throughout the study. Participants perceived the program to be useful in improving emotional awareness and eating control. Program feasibility was supported by the retention rate (70%) and the average number of attended sessions (*M* = 9.71; *SD* = 2.06). Further studies are needed to examine the effectiveness of EF-CBT interventions.

## Introduction

Obesity is a major public health problem associated with an increased risk of chronic illness and mortality (James, 2008). It is estimated that at least 2.8 million adults die each year as a result of excessive weight (Ellulu et al., 2014). Binge eating disorder (BED) is an important co-occurring problem with obesity, consisting of persistent episodes of excessive food intake, accompanied by a sense of lack of control, and occurring without recurrent inappropriate compensatory behaviors (American Psychiatric Association [APA], 2013). The BED prevalence among overweight and obese samples seeking weight loss treatment varies between 20 and 30% (Striegel-Moore and Franko, 2003). However, recent data suggest that comorbid obesity and BED is increasing at a faster rate than obesity alone (Darby et al., 2009; da Luz et al., 2017). Moreover, weight loss has proven to be significantly greater in patients with obesity who achieved and sustained binge remission in the long-term (Palavras et al., 2017). These findings suggest that these two problems, which are often studied and treated separately, should be addressed together. In spite of this, psychological therapies have mostly been borrowed by general obesity management (Amianto et al., 2015) and do not explore the relationship between these two conditions. Effective integrated approaches to obesity and BED that relieve the dysfunction due to overeating while enhancing appropriate weight loss are required (Palavras et al., 2017).

A possible clinical approach to comorbid obesity with BED is targeting emotions related to eating behavior. It has been hypothesized that emotional distress may increase the use of overeating as a strategy to regulate or escape from negative affect (e.g. Heatherton and Baumeister, 1991; Stice et al., 2001; Levitan and Davis, 2010). The tendency to eat in response to negative emotions is defined as emotional eating (Arnow et al., 1995). Theoretically, emotional eating promotes the consumption of large amounts of food and, consequently, the development of binge eating (Haedt-Matt et al., 2014). Empirical data in patients with comorbid obesity and BED also support this link between emotions and eating. It was found that difficulties regulating emotions were strongly associated with emotional overeating (Gianini et al., 2013). Moreover, a recent systematic review and meta-analysis concluded that individuals with obesity, especially those with comorbid BED, revealed lower levels of emotional awareness and difficulty using emotion regulation strategies (Fernandes et al., 2017). Binge eating behavior can emerge to compensate for emotion regulation difficulties in obesity, but it can itself potentiate emotion regulation difficulties by limiting the use of more efficient strategies (Leehr et al., 2015). Therefore, it is relevant to address emotional aspects related to eating in the treatment of obesity, especially in patients with BED comorbidity.

To date, Cognitive Behavioral Therapy (CBT) is recognized as the best established treatment for obesity (for review see Brennan et al., 2014; Castelnuovo et al., 2017; Jacob et al., 2018) and obesity coupled with BED (for review see Duchesne et al., 2007; Palavras et al., 2017). In regard to emotional eating, the efficacy of CBT has been revealed to be superior to other interventions [cf. review of Jacob et al. (2018)]. However, the low magnitude of the differences at the emotional level together with the modest efficacy in maintaining long-term weight loss (Cooper et al., 2010) and binge eating reduction (Juarascio et al., 2017; Palavras et al., 2017), indicate the need for improved interventions. In acknowledgment of the importance of emotions in obesity, new CBT programs have put greater emphasis on emotion regulation training, such as impulsive eating (Preuss et al., 2017), food cravings (Stapleton et al., 2017), emotional functioning (Buckroyd et al., 2006; Buckroyd and Rother, 2007), and emotion regulation skills (Pjanic et al., 2017). In general, these new CBT programs found encouraging results in terms of emotional functioning (Buckroyd et al., 2006; Pjanic et al., 2017; Stapleton et al., 2017), eating behavior (Preuss et al., 2017; Stapleton et al., 2017) and weight loss (Buckroyd et al., 2006; Preuss et al., 2017). However, with exception to Buckroyd et al.’s (2006) program, these new emotion-focused CBT interventions did not target individuals with obesity that eat compulsively. Although a greater focus on emotional processes may be a means to improve treatment outcomes in obesity with comorbid BED (Gianini et al., 2013), there is little knowledge about the feasibility and acceptability of this approach. Previous studies concerning CBT, both traditional and “third generation” interventions, have mainly focused on efficacy (as documented in review studies, e.g. Duchesne et al., 2007; Forman et al., 2015; Alimoradi et al., 2016), with little attention given to the experience of receiving the treatment. Whether it is acceptable, safe, and beneficial for this target population remains unclear. Therefore, this study aimed to determine the feasibility of a brief emotion-focused CBT (EF-CBT) program for women with comorbid obesity and BED, exploring attrition and intervention acceptability. We also sought to gather preliminary data on long-term outcomes, considering the need to improve long-term effectiveness of the treatment.

Attrition was analyzed in terms of retention and attendance rates. Based on previous weight-loss interventions (Miller and Brennan, 2015), we predicted that 70% of the sample would complete the intervention, and those who completed it would attend, on average, a minimum of eight sessions, corresponding to 65% of the program. No serious adverse events were expected and we predicted that participants will perceive the therapy to be helpful overall. Lastly, we hypothesized that outcome variables, mainly those related to emotion processing abilities, will improve after the intervention, including successive follow-ups. Regarding weight loss, we expected to achieve a 5–10% reduction in initial weight (National Institutes of Health, 1998).

## Materials and Methods

### The EF-CBT Program

The EF-CBT program is an extension of CBT for weight management (Cooper et al., 2003), enriched with emotion regulation strategies adapted from the Buckroyd and Rother’s (2007) approach. The overall aim of the intervention was to improve emotional processing and reduce maladaptive eating patterns that have an important impact on weight management. The EF-CBT program was designed to be effective in terms of cost and time, avoiding the sustainability problems found in previous programs (Brennan et al., 2014). Sessions were reduced to the bare minimum and the intervention was carried out in a group format, enabling a greater number of individuals to receive the treatment. The program was structured into 12 weekly sessions, with 90 min per session, for 3 months. The group was taken through four stages, covering a range of themes. *Stage 1* involved explorations of personal history and weight background, including causal attributions of obesity, obesity myths and beliefs, and obesity stigma. *Stage 2* explored binge eating patterns and related contingencies. It places emphasis on self-awareness of eating behavior and the counterproductive effects of restrained eating. *Stage 3* focused on the nature and function of emotions and their relationship to eating behavior, body image and self-esteem. Following the previous stage, it was explored the role of emotions and how to identify and express them. *Stage 4* addressed emotion regulation strategies that are alternatives to emotional eating. Emotional and cognitive-behavioral strategies were applied in order enable the participants to deal with emotional triggers of overeating. Examples of strategies used are: relaxation, assertiveness and social skills training, rational analyses, evidence testing, body metaphors, role-playing, and emotion and thought registration.

The group was facilitated by a qualified clinical psychologist (principal facilitator) who had extensive experience in obesity and group therapy and was co-facilitated by a clinical psychologist trainee. Neither professional was part of the research team and worked at the Outpatient Center Clinic of the Faculty of Psychology and Education Sciences, University of Porto (FPCEUP).

This longitudinal pilot study is part of a larger project entitled “Outcome and change process research on emotion-focused approaches in obesity” carried out by the FPCEUP in partnership with the Centro Hospitalar São João, Porto. The research was conducted in accordance with the Declaration of Helsinki, and the protocol was approved by the Ethics Committee of Centro Hospitalar São João (reference number: CES 42-2011). All subjects gave their informed consent for inclusion before they participated in the study.

### Participants

Participants were recruited through flyers and internet advertisements to join an emotion-focused program for obesity that will be held at the Outpatient Center Clinic of the FPCEUP. This pilot study was conducted with female participants only, because they are more representative of the population seeking weight-loss treatment. Inclusion criteria were older than 18 years old, female gender, body mass index (BMI) equal to or greater than 30 kg/m^2^, BED diagnosis (according to DSM-5; American Psychiatric Association [APA], 2013), and currently not under other treatment (medical, psychological, or nutritional). Exclusion criteria included illicit substance use or alcohol abuse and dependence, psychotic comorbidity, and prior weight loss surgery. Comorbid BED diagnosis was established through the application of the Interview for the Diagnosis of Eating Disorders-IV (IDED-IV; Kutlesic et al., 1998; Torres et al., 2008; Pereira, 2009). Ten women were considered eligible and attended the first session. Their mean age was 47.3 years (*SD* = 18.82) and mean BMI was 32.4 kg/m^2^ (*SD* = 3.37). Three of these participants dropped out from the group; two dropped after session 1 and one after session 6. We did not receive any reply when contacting the two participants who dropped out after session 1. The other participant dropped out at session 6 due to moving to another city.

We present data corresponding to the seven participants who completed the program. From those, one participant did not complete the 6- and 18-month follow-up assessments. At baseline the seven participants were aged between 26 and 56 years old (*M* = 38.29; *SD* = 9.32) and the BMI varied between 30 and 38.86 kg/m^2^ (*M* = 34.61; *SD* = 3.91).

### Material

#### Anthropometric Measurement

Height and weight were obtained by direct measurement with SECA digital scale and stadiometer, to calculate the BMI (kg/m^2^). BMI equal or greater than 30 kg/m^2^ was indicative of obesity diagnosis.

#### Interview for the Diagnosis of Eating Disorders-IV

The IDED-IV (Kutlesic et al., 1998; Torres et al., 2008; Pereira, 2009) is a semi-structured interview for differential diagnosis of eating disorders based on DSM-IV-TR criteria (American Psychiatric Association [APA], 2000). With the transition to DSM-5 (American Psychiatric Association [APA], 2013), the BED was recognized as a distinct diagnostic category and its operational definition was slightly changed. The frequency threshold for binge eating changed from at least twice weekly for 6 months (as in DSM-IV-TR) to at least once weekly over the three most recent months (as in DSM-5). We adjusted this threshold when assessing the presence of this symptom.

#### Clinical Outcomes in Routine Evaluation-Outcome Measure

The CORE-OM (Evans et al., 2000; Sales et al., 2012) is a 34-item measure of general psychological distress in adults, covering four domains: (1) Subjective well-being; (2) Problems/symptoms, including depression, anxiety, physical aspects, and effects of trauma; (3) Functioning, including close relationships, general functioning, and social aspects; and (4) Risk, including risk to self and risk to others. Each item is rated on a 5-point scale ranging from 0 (*not at all*) to 4 (*most or all the time*). Higher scores indicate more psychological distress. The clinical cut-off mean score for the CORE-OM is 1.

#### Simplified Personal Questionnaire

The PQ (Sales et al., 2007c; Elliott et al., 2008) is an individualized change measure, consisting of roughly ten problems in the participant’s own words, that bother him/her the most and that s/he wishes to address in therapy (Sales and Alves, 2012). The questionnaire contains a semi-structured interview that helps patients to state their main psychological difficulties. The written statements are placed on individual note cards, rank-ordered and typed onto a standard form. Patients rate the intensity of distress caused by each problem (“how much has it bothered you during the past 7 days, including the present day”), using a 7-point scale (from *not at all* to *maximum possible*). Patients can change it by entering new complaints or deleting previous items if they wish. However, in this study, we only analyzed the problems that were reported at baseline and which have remained post-intervention. The clinical cut-off point for the PQ is 3.25 (Elliott et al., 2016).

#### Dutch Eating Behavior Questionnaire

The DEBQ (van Strien et al., 1986; Viana and Sinde, 2003) is composed of a 33-item measure assessing emotional eating (occurrence of overeating as a way to cope with emotions), external eating (eating in response to the sight or smell of food), and restrained eating behavior (pattern of dietary restraint that is followed by increased consumption and binge eating). Each item is rated on a 5-point scale ranging from 1 (*never*) to 5 (*very often*). Higher scores mean a greater tendency toward maladaptive eating behavior patterns.

#### Emotional Processing Scale

The EPS (Baker et al., 2010; Leitão and Torres, 2015) is a 25-item questionnaire designed to identify difficulties in the processing of emotions, which is also recommended to measure change in emotional processing dimensions during therapy. The EPS has a five-factor structure, with five items per factor: (1) Suppression (excessive control of emotional experience and expression), (2) Signs of unprocessed emotion (intrusive and persistent emotional experiences), (3) Unregulated emotion (inability to control emotions), (4) Avoidance (avoidance of negative emotional triggers), and (5) Impoverished emotional experience (experience of emotions due to poor emotional insight). Responses are given on a 0–9 Likert scale. Higher scores are indicative of poorer emotional processing.

#### Toronto Alexithymia Scale – 20 Items

The TAS-20 (Bagby et al., 1994; Prazeres et al., 2000) is a 20-item measure covering three main areas of the alexithymia construct: (1) difficulty in identifying feelings (DIF), (2) difficulty in describing feelings to others (DDF), and (3) externally oriented thinking style (EOT). Each item is rated on a 5-point Likert scale ranging from 1 (*strongly disagree*) to 5 (*strongly agree*). Higher scores mean a greater tendency toward alexithymia. Total scores of 61 and higher are considered to be indicative of alexithymia.

#### Helpful Aspects of Therapy

The HAT (Llewelyn, 1988; Sales et al., 2007b) is a post-session self-report questionnaire that addresses patients’ perceptions of key change processes in therapy (Sales et al., 2007a). Patients are asked to identify and describe in their own words significant events that happened from session to session. The positive events identify possible beneficial effects of therapy and negative events or those that hinder therapy were considered as possible adverse effects of therapy.

#### Client Change Interview

The CCI (Elliott et al., 2001; Sales et al., unpublished) is an interview with four to eight open-ended questions that is administered when the therapeutic process ends. The CCI provides a qualitative overview of the changes that a person has noticed since beginning therapy, what the person attributes these changes to, and helpful and unhelpful aspects of therapy (Elliott, 2010). The participant is asked to identify changes that she has noticed and then asked to rate each of these changes (from 1 to 5) in terms of expectations toward change, therapy’s impact on change, and the importance of the change.

### Procedures

#### Data Collection

Standardized outcome measures (CORE-OM, DEBQ, EPS, and TAS-20) were administered at four assessment times: baseline, post-intervention, and at the 6- and 18-month follow-ups. The individualized outcome measure PQ was administered at baseline and post-intervention. The HAT form was applied at the end of each session, asking patients to report their experiences concerning the session. The CCI was conducted at post treatment, 6- and 18-month follow-up. These measures were applied by a research team member and were completed in the Outpatient Center Clinic.

#### Data Analysis

We analyzed change in four main domains: weight loss, general distress (CORE-OM and PQ), eating behavior (DEBQ), and impaired emotional functioning (EPS and TAS-20). Weight loss was expressed by the percent baseline weight loss [%WL = (weight lost/baseline weight) ^∗^ 100], a commonly used metric in behavioral literature (Hatoum and Kaplan, 2013).

Due to the study being a pilot and having a small sample size, data analyses were mainly descriptive and exploratory. No inferential statistical comparisons were made. Magnitude of the changes in the means for the four point-in-time assessments was estimated using partial eta square (η*_*p*_*^2^) or Cohen’s *d*. We used the benchmarks proposed by Cohen (1988) to define small (η*_*p*_*^2^ = 0.01; *d* = 0.2), medium (η*_*p*_*^2^ = 0.06; *d* = 0.5), and large (η*_*p*_*^2^ = 0.14; *d* = 0.8) effects.

For a better understanding of the therapeutic effects we determined the reliability of changes for each participant in outcome measures, through the Reliable Change Index (RCI). An RCI indicates whether the change in an individual’s score over time is greater than might would be expected from random variation alone. The RCI can be defined as the patient’s score change (difference score between two measurements), divided by the standard error of the difference. Values larger than 1.96 are seen as statistically significant at a two-tailed 5% alpha level. The following parameters were considered: RCI greater than 1.96 is defined as improvement; RCI less than −1.96 is defined as deterioration; and RCI values between −1.96 and 1.96 are defined as absence of change (Jacobson and Truax, 1991).

We also analyzed the clinical significance of change as an additional indicator. Changes were considered clinically relevant when moved from a clinical to non-clinical range. As this analysis requires the use of normative cut-off values, we were only able to perform it for TAS-20, CORE-OM, and PQ.

The PQ items were content-analyzed by two independent raters. Inter-rater reliability measured by Cohen’s kappa (κ) was 0.84. The content analysis of the problems identified by patients resulted in seven categories. We also calculated the mean score of the intensity of distress caused by all complaints together at baseline and post-intervention. Cohen’s *d* was calculated to provide effect size for the differences between these assessment times.

Feasibility of the program was assessed by (1) retention and attendance rates, and (2) adverse and beneficial aspects of the program reported by patients in the HAT and CCI. Qualitative data provided by the HAT and CCI were also content-analyzed by two independent raters to derive meaning and to identify recurring conceptual patterns of experience, organizing the information by categories (Elliott and Timulak, 2005). For HAT, the significant events were categorized from the adaptation of a grid of categories of significant events in psychotherapy, available in the literature (Timulak, 2007, 2010; Elliott, 2010; Castonguay, 2011; Richards and Timulak, 2012; Vieira, 2014). The codification in each category was not mutually exclusive. The kappa coefficient for HAT was 0.69. Frequencies of responses in each category were analyzed. In CCI, patients identified a broad diversity of changes that were organized into eight categories. The kappa coefficient was 0.91.

## Results

### Weight and Psychological Changes

A favorable evolution in the mean scores for weight was observed. Comparing with the baseline, the mean %WL at the post-intervention, 6-month and 18-month follow-ups was 4.57% (*SD* = 4.26), 6.98% (*SD* = 5.99), and 4.10% (*SD* = 5.87), respectively.

Table 1 displays descriptive statistics concerning average scores on the psychological measures, along with estimated effect sizes for differences over time-point assessments. Positive long-term changes, with large effect size (η*_*p*_*^2^ between 0.160 and 0.593), were found in all outcome variables, with exception to restrained eating (η*_*p*_*^2^ = 0.032). There was a trend toward an improvement in average psychological distress, emotional processing, and alexithymia both in post-intervention and 6-month follow-up. At the 18-month follow-up, this trend was no longer observed but the mean scores still remained more satisfactory than for the baseline. Only three subscales of these measures registered a different pattern: (1) the CORE-OM-Risk, for which the improvement was only observed at the 18-month follow-up; (2) the TAS-Externally oriented thinking style, whose improvement was mainly noted at post-intervention; and (3) the EPS- Impoverished emotional experience, which has increased at the 18-month follow-up to higher levels than the baseline, countering the decreasing tendency observed at previous points in time. In eating behavior, changes in emotional and external eating over time were of large effect (η*_*p*_*^2^ = 0.450 and η*_*p*_*^2^ = 0.478, respectively); we found lower mean scores at the 18-month follow-up relative to the baseline. However, in contrast to most outcome variables, external eating reduced steadily over time, not slowing down at the 18-month follow-up. Restrained eating was the only outcome variable with no changes in post-intervention and follow-up periods (η*_*p*_*^2^ = 0.032).

**TABLE 1:** Change in outcome variables over the four assessment times.

	Baseline	Post-intervention	6-month follow-up	18-month follow-up		
				
	*M* (*SD*)	*M* (*SD*)	*M* (*SD*)	*M* (*SD*)	η*_*p*_*^2^	*d*
**General distress**						
PQ						
Intensity^b^	5.51 (0.71)	4.14 (1.51)				0.890
CORE-OM						
Well-being	1.87 (0.98)	1.45 (0.94)	1.00 (0.63)	1.33 (0.56)	0.593	
Problems/symptoms	1.75 (1.14)	1.31 (0.86)	1.14 (0.85)	1.49 (0.72)	0.302	
Functioning	1.36 (0.92)	1.08 (0.85)	0.95 (0.69)	1.04 (0.63)	0.417	
Risk	0.17 (0.33)	0.22 (0.39)	0.17 (0.28)	0.08 (0.14)	0.160	
Total	1.35 (0.87)	1.05 (0.76)	0.89 (0.65)	1.06 (0.54)	0.387	
**Eating behavior**						
DEBQ						
Emot Eat	3.73 (1.45)	2.89 (1.05)	3.17 (1.37)	3.10 (1.29)	0.450	
Ext Eat	3.26 (0.92)	2.83 (0.73)	2.56 (0.76)	2.45 (0.67)	0.478	
Rest Eat	3.12 (0.79)	3.28 (0.40)	3.18 (0.33)	3.08 (0.67)	0.032	
**Emotional processing**						
EPS						
Suppression	4.83 (2.16)	3.47 (2.62)	2.70 (2.19)	2.77 (2.24)	0.343	
Signs Unp. Emot	4.80 (2.60)	4.00 (2.21)	2.70 (2.09)	3.00 (2.62)	0.507	
Unreg emotion	3.43 (1.65)	1.93 (1.41)	1.83 (1.13)	3.40 (2.57)	0.337	
Avoidance	4.53 (1.35)	3.20 (1.92)	2.53 (1.34)	3.00 (2.22)	0.333	
Imp emot exper	2.93 (1.48)	2.10 (1.15)	2.07 (1.18)	3.27 (2.15)	0.374	
Total	4.10 (1.48)	2.94 (1.59)	2.36 (1.34)	3.08 (2.11)	0.472	
TAS-20						
DIF	20.83 (8.75)	16.83 (6.71)	15.00 (5.18)	18.00 (7.67)	0.403	
DDF	12.17 (4.31)	12.00 (6.00)	11.83 (5.46)	10.83 (4.31)	0.103	
EOT	18.83 (5.67)	16.83 (6.31)	17.17 (3.87)	17.33 (5.96)	0.189	
Total	51.83 (16.04)	45.66 (17.73)	44.00 (12.75)	46.16 (17.2)	0.414	

**PQ – Simplified Personal Questionnaire; CORE-OM – Clinical Outcomes in Routine Evaluation-Outcome Measure; DEBQ – Dutch Eating Behavior Questionnaire; Emot Eat – Emotional Eating; Ext Eat – External Eating; Rest Eat – Restrained eating; EPS – Emotional Processing Scale; Signs Unp. Emot. – Signs of Unprocessed Emotions; Unreg emotion – Unregulated emotion; Imp emot exper – Impoverished emotional experience; TAS-20 – Toronto Alexithymia Scale – 20 items; DIF – difficulty in identifying feelings; DDF – difficulty in describing feelings to others; EOT – externally oriented thinking style. ^*b*^Discomfort intensity considering all complaints together.**

Participants reported a total of 23 individualized complaints in the PQ, which included: weight concerns (*n* = 6); negative view of self (e.g. low self-esteem; *n* = 5); family and relational concerns (*n* = 3); academic/work/economic concerns (*n* = 3); health concerns (*n* = 2); psychological symptoms (e.g. anxiety, depression; *n* = 2); and functionality impairment (e.g. lack of energy, lack of daily planning or discipline; *n* = 2). The mean intensity of distress caused by all complaints together decreased at the end of the intervention, with a large effect size (*d* = 0.890).

Rates of reliable change are reported in Table 2. Reliable improvement was most common for complaints (*n* = 4), psychological distress (*n* = 4), external eating (*n* = 3), emotional processing (*n* = 7), and alexithymia (*n* = 6). With the exception of alexithymia (*n* = 1), no participants registered a deterioration in these variables. Participants’ improvement in these outcome measures was often continuous, occurring along the three time-points. In contrast, reliable positive changes in emotional (*n* = 3) and restrained eating (*n* = 2) were more modest. In addition, a total of three participants registered a decline in these eating styles during the study.

**TABLE 2:** Rates of reliable change in outcome measures at post-intervention, 6-month and 18-month follow-ups.

	RCI		
	
Variable	Baseline – post	Baseline – 6-months	Baseline – 18 months
**PQ**			
Improvement	P1, P2, P3, P6	–	–
Deterioration	None	–	–
**CORE-OM**			
Improvement	P1, P2, P4, P7	P1, P2, P4, P7	P1, P7
Deterioration	None	None	None
**DEBQ – emotional eating**			
Improvement	P1, P3, P5	P1, P3	P1, P3
Deterioration	P2	P2	P2, P7
**DEBQ – external eating**			
Improvement	P1, P3, P5	P1, P3, P5	P1, P3, P5
Deterioration	None	None	None
**DEBQ – restrained eating**			
Improvement	P5	P1, P5	P1, P5
Deterioration	P2, P3	P3	P2
**EPS**			
Improvement	P1, P2, P3, P5, P6, P7	P1, P2, P3, P4, P5, P7	P1, P2, P3, P4
Deterioration	None	None	None
**TAS-20**			
Improvement	P1, P2	P1, P2, P5, P7	P1, P2, P3, P4, P7
Deterioration	P6	None	None

*PQ – Simplified Personal Questionnaire; CORE-OM – Clinical Outcomes in Routine Evaluation-Outcome Measure; DEBQ – Dutch Eating Behavior Questionnaire; EPS – Emotional Processing Scale; TAS-20 – Toronto Alexithymia Scale – 20 items. P0 – participant 0. Improvement = RCI ≤ −1.96. Deterioration = RCI ≥ 1.96.*

In the analysis of the clinical relevance of changes, we observed that, at the baseline, some participants scored above the clinical cut-off points for psychological distress (*n* = 4) and alexithymia (*n* = 2). The intensity of distress caused by psychological difficulties listed in the PQ was clinically relevant for all participants (*n* = 7). Recovery (i.e. moving from clinical to non-clinical range) post-intervention was only found in the PQ, for two participants. Although psychological distress (*n* = 3) and alexithymia (*n* = 1) has decreased in some participants, post-intervention and follow-up times, this improvement did not reach clinical significance.

### Program Feasibility

Of the 10 participants included, 7 fulfilled the EF-CBT program, which resulted in a completion rate of 70%. Of those who did not complete the intervention, only one had participated in more than one session. The average number of attended sessions for those completing therapy was 9.71 (*SD* = 2.06; range = 6–12). One participant did not complete the 6- and 18-month follow-up assessments.

Over the course of the 12 sessions, participants identified 92 positive/beneficial events, and four hindering/adverse events, organized in seven thematic categories (Table 3). The most helpful aspects of the sessions were the promotion of insight/self-awareness/self-knowledge (36%), positive experiences derived from group factors (19%), and from specific techniques or program characteristics (16%). Negative experiences corresponded to only 4% of the total events, mostly related to inhibition and discomfort in the early sessions.

**TABLE 3:** Helpful aspects of therapy: description and illustrative quotes of significant positive and hindering events.

	Description	Illustrative quotes
**Positive events (*n* = 92)**		
Insight/Self-awareness/Self-knowledge (*n* = 35)	The individual becomes more aware of his/her feelings, cognitions and behaviors. Developing a clearer understanding of himself/herself, others and situations. Development of a new perspective on one’s personal reality.	It was positive because it made me see that there are so many things that ultimately had no importance, and there are others that never get to talk (p1s2). One of the comments made me wonder if my excess weight is not an escape from unwanted looks (p5s4)
Empowerment/Positive Self-perception (*n* = 6)	Development of personal competence to deal with problematic situations. Feelings of strength, development and personal value. Positive expectations and evaluations of oneself.	You can do it (p2s5) Realize that I am much more than my weight and I have everything I want in life (p1s12) Gaining awareness of who I am (p7s10)
Problem clarification (*n* = 13)	The individual relates a clearer understanding of his/her problems or what he/she wants to change; What goals to achieve becomes clear to the individual, how to handle situations.	Explain the behavior that mostly influenced the process of getting fat (p3s3) Realizing what is really important, what are priorities (p5s12)
Emotional experience (*n* = 5)	The individual stresses the externalization of feelings as the most significant aspect in the session. Experience “here and now” new or old emotions, in a new perspective.	I have talked about my feelings and I have cried (p3s7) It is good to speak out on a matter so delicate (p1s1)
Program characteristics (*n* = 15)	Positive overall assessment of the session/positive appraisal techniques in the session	The way the session was conducted broke the ice and facilitated the creation of the group (p6s1) Making the Road Map (p7s2) Relaxation (p3s11)
Group factors (*n* = 18)	Cohesion feelings, support, safety and well-being related to the group. Feeling understood by the group; relativizing problem by comparison with another; feelings of group identity, universality and mutual aid.	The host of the group in terms of feeling heard; the openness to change by all (p3s1) It was very interesting to talk of dietary mistakes by sharing ideas that lead people to become more aware (p6s3) Realize that there are those who think and feel the same as we do (p2s6)
**Hindering events (*n* = 4)**		
Unwanted thoughts/feelings (*n* = 4)	Discomfort with the experiences that occurred in the session, particularly something that has been said or something that was experienced.	Not being very comfortable when I must talk about my own feelings (p4s1) I feel at this point that I will not come again (p1s8)

*p0s0 – participant 0, session 0.*

In post-intervention, participants identified a total of 27 changes (Table 4) that were retrospectively attributed to the program. The most frequent changes were higher awareness of eating behavior triggers and consequences (26%), increased eating self-control (18.5%) and improvements in lifestyle (18.5%). Results in both follow-ups were slightly different. At the 6-month follow-up, among the 19 changes identified, the most salient were the positive relational changes (36.8%) and the new category weight loss (21%). At the 18-month follow-up, with 15 changes reported, positive relational changes (26.6%) remained as the most highlighted, and improvement in body-esteem (26.6%) became more prominent.

**TABLE 4:** Changes identified in the CCI at post-intervention, 6- and 18-month follow-up.

Changes	Post-intervention (*N* = 27) *n* (%)	6-month follow-up (*N* = 19) *n* (%)	18-month follow-up (*N* = 15) *n* (%)	Illustrative quotes
Awareness of binge eating triggers and consequences	7 (26.0)	2 (10.5)	3 (20.0)	Increased awareness of the moments when I eat excessively (p4); Awareness of the relationship between emotion and food (p7).
Greater eating self-control	5 (18.5)	0	2 (13.3)	I feel more capacity and self-control over food (p7); Reduction of binge eating episodes (p5).
Lifestyle improvement	5 (18.5)	2 (10.5)	0	Smoke and drink less (p2); I started exercising (p1).
Positive emotional changes	3 (11.1)	1 (5.3)	1 (6.7)	I feel more serenity, calm (p1); I stopped valuing some aspects that are out of control (p1).
Positive influence on others	3 (11.1)	7 (36.8)	4 (26.6)	My boyfriend becomes more attentive to food (p3); Influence on my mother’s diet (p6).
Body-esteem improvement	2 (7.4)	2 (10.5)	4 (26.6)	Discover the vanity, take care of myself for me and not for others (p3); I feel better regarding my body (p2).
Increased difficulties	2 (7.4)	1 (5.3)	0	More difficult to look at the mirror (p4); Heightened sense of claustrophobia (p2).
Weight loss	0	4 (21.0)	1 (6.7)	I lost weight (p5).

When asked about the expectations regarding these changes, 66.7% of the participants mentioned that they were *somewhat* or *totally surprising*. Most of the unexpected changes were related to the awareness about eating behavior triggers and consequences, awareness of emotions and their relationship with eating behavior, and body-esteem improvement. The degree of importance attributed to these changes was *very meaningful* and the majority of participants (63%) considered that changes would not have happened without the intervention.

## Discussion

This study explored the feasibility and long-term outcomes of a brief CBT group intervention targeting overeating and underlying emotions with an impact on weight management. Globally, retention and attendance rates met the pre-specified targets for determining feasibility. No serious adverse events were noted and participants perceived the therapy as being helpful. The findings suggest the program’s long-term efficacy in improving psychological distress, emotional processing, and alexithymia in women with obesity and BED. Positive reliable changes in emotional processing and alexithymia were observed in almost all participants. These changes occurred at all time-points, despite mean scores at the 18-month follow-up that suggest a slowdown tendency. Participants did not report the loss of any previous change, suggesting that improvements were maintained over time. Weight loss was less than what was expected for all time-points, as well as changes in emotional and external eating. Restrained eating mean scores remained stable throughout the study.

It is widely accepted that obesity affects people’s quality of life, and improvements in patients’ well-being and psychosocial functioning are considered relevant indicators of treatment efficacy (Mannucci et al., 2010). The reliable clinical changes in psychological distress (CORE-OM) achieved by four participants suggested the presence of these gains. Qualitative data is also in line with this view; participants identified positive changes in lifestyle, body image and self-care as benefits of therapy.

Nevertheless, quantitative data indicates that emotional processing and alexithymia were the more strongly impacted outcome measures. This is not a surprising result, considering the goals and content of the EF-CBT approach. A close inspection of the subscales revealed that changes of a larger magnitude were relative to the EPS-Signs of Unprocessed Emotion, EPS-Suppression, EPS-Avoidance, and TAS-Difficulty in identifying feelings. Overall, these findings suggest there is potential for the EF-CBT program to address unprocessed emotional material. From the theoretical point of view, this process may have been facilitated by an increase in emotional awareness and expression – two key dimensions in the alexithymia construct (Bagby et al., 1994). There is evidence that emotional difficulties observed in patients with obesity are rooted in an emotion avoidance process. It is possible that this emotional avoidance style develops as a way to deal with stressful experiences, likely due to weight stigmatization (Fernandes et al., 2017). In this context, increasing awareness of emotion, or naming what one feels, can be a key feature for enabling adaptive emotional responses. Becoming aware of emotional experience provides access both to adaptive information and to the tendency in emotions related to action (Greenberg, 2010). The impact of the EF-CBT program on the emotional domain was also reinforced by the qualitative feedback of the participants, with insight and self-awareness emerging as the most prominent positive events from therapy. Moreover, clarification concerning emotional states and their relationship with eating behavior were also reported by participants after the therapy, which shows that intervention has the potential to promote the understanding of feelings, cognition and behaviors.

Regarding eating behaviors, we found reduced mean scores in external eating and emotional eating for all time-points, when compared to the baseline. Three participants recorded, simultaneously, a positive reliable change in these two variables. It is theoretically plausible that the intervention may have had a similar effect on these eating styles, because they tend to co-occur (van Strien, 2018). Emotional eaters often shift their attention away from negative emotions by driving attention to appetizing food cues, enabling external eating. According to empirical research, emotional distress seems to sensitize the brain’s reward system to appetitive stimuli, precipitating self-regulatory failure and, in turn, overeating (Wagner et al., 2012). Thus, external and emotional eating, as interwoven expressions of overeating, can each benefit from the decrease in alexithymia and emotional processing deficits, giving place to the development of alternative coping strategies to deal with emotions. With this in mind, we consider the participants’ perception of increased eating self-control reported in the interview (at post-intervention and 18-month follow-up) to be coherent with the reduction of external and emotional eating. Also congruent with the positive evolution of emotional eating is an increased awareness of binge eating triggers – another change noted by most of the participants at post-intervention interview. Being aware of the overeating episodes and their relationship with emotions could have facilitated the reduction of uncontrolled eating (van Strien, 2018).

However, it should be noted that positive reliable changes in emotional eating were only observed in three participants and two others reported a deterioration. This finding leads to more modest results than expected. In particular, the occurrence of negative changes deserve consideration. A close look at the reliable changes of these two participants shows that they both have improved on emotional processing and alexithymia. In our view, it is possible that an increased predisposition to feel emotions together with an improved ability to recognize them, may have brought to consciousness a set of suppressed difficult emotions. In fact, self-awareness relative to several areas, such as personal difficulties, body image, and dysfunctional relationships, was the useful aspect of therapy most frequently indicated by the participants. This process could have elicited intense negative emotions such that, in the face of difficulties of self-regulation, individuals have found in food an emotional compensation. There is empirical evidence that self-regulating eating to overcome emotional cues can decrease in efficacy given the presence of anxious-depressive symptoms (Annesi, 2020) and body dissatisfaction (Annesi and Mareno, 2015). To prevent these cases, the EF-CBT should be enriched with more attention on self-efficacy and self-regulatory skills.

Despite the encouraging findings regarding emotional processing achieved in this study, we should keep in mind that there is still room for progress. There is no evidence of improvement in subscale scores of the EPS relative to unregulated emotions and impoverished emotional experience at the end of the study. Even considering that some participants may have scored within the normal range at baseline (floor effect), it is possible that some emotion regulation strategies need to be strengthened, because they are difficult to develop and consolidate in a short-term intervention, as previously stated by Vieira (2014). To achieve this goal, a few individual follow-up sessions could be conducted to address specific difficulties. They could also enable the consolidation of the changes achieved, preventing their long-term loss from focus.

The future challenge for the EF-CBT program is, thus, to improve the effect on emotional eating, on the assumption that it is a stronger predictor of weight change (Koenders and van Strien, 2011). From this point of view, the modest weight reduction observed at the end of the study (4.10% WL) is theoretically congruent with the lower-than-expected finding in emotional eating. However, it should be noted that this result, despite not reaching the established goal, is not substantially different from those observed in behavioral treatment programs. A meta-analysis of lifestyle modification interventions found that, 1 year after intervention, only 28% of individuals had a weight loss of ≥10% of baseline weight, and a substantial percentage of participants (38%) had a weight loss of ≤4.9% (Christian et al., 2010). In addition, we must take into consideration that 4 of the 7 study participants had an initial BMI between 30 and 33 kg/m^2^, indicating that they needed to lose a smaller amount of weight. Idiographic data on this issue are quite encouraging. The participants perceived a decrease of the discomfort caused by weight concerns, as registered in the PQ, as well as weight loss as an emerging change resulting from the therapy. Nevertheless, it seems clear that the effect of the EF-CBT intervention on weight reduction needs further improvement.

In our view, better progress in this variable can be promoted by a significant decrease in emotional eating, as mentioned above, but also by expressive improvements in other eating behavior dimensions. It is known that external and restrained eating have an adverse effect on appetite control (Burton et al., 2007) and, for this reason, deserve consideration. In our study, changes in appetitive response to external cues were in a positive direction but, in practice, only three participants showed reliable improvements. In turn, restrained eating improved in two participants, but deteriorated in two others. Interestingly, deterioration in one participant was concomitant with deterioration in emotional eating. A bidirectional relationship between these dimensions of eating behavior is plausible, since food restriction can be a strategy to compensate for emotional overeating, and overeating can emerge as a way to counteract the effects of self-imposed restrictions on food intake (Grilo et al., 2001; Stice et al., 2001; Snoek et al., 2007). Therefore, we propose to reinforce a greater focus on the counterproductive effect of skipping meals and restricting food intake in the program content, as well as to raise awareness of the reasons behind these practices.

Program content can also be improved by exploring the difficulties associated with long-term weight loss maintenance. In this study we observed a downward trend in average the weight-loss percentage between the 6-month and 18-month follow-ups (from 6.98 to 4.10%), evidencing the difficulty sustaining a new lower weight. Long-term weight management is challenged by a complex interaction of environmental, biological, and behavioral factors. In the context of these factors, it could be pertinent to add a weight-maintenance training element to the program that includes strategies to help individuals anticipate struggles and prepare contingency plans, moderate behavioral fatigue, and put the inevitable lapses into perspective. Long-term vigilance is also needed (Hall and Kahan, 2018) and can be integrated in the individual follow-up sessions proposed above.

Our findings confirmed the high acceptability of the EF-CBT program, both in terms of content and intervention format. Indeed, the intervention content had been designed with the aim of promoting emotional awareness, self-disclosure, and emotional expression, which were the most significant aspects of the intervention for the participants. In regard to the intervention format, we should stress the importance of group factors reported by the participants. As described by Yalom and Leszcz (2005), the inevitable exchange of information in a group setting helps members to reflect on personal experiences. Group cohesiveness provides a sense of acceptance and security, which motivates self-disclosure. By disclosing information to the group, participants can release suppressed emotions (Catharsis). In addition, group members realize they are not alone in their problems (Universality) and this therapeutic factor may serve as a motivator to change processes. As a result, the commitment to therapy can be strengthened.

Accordingly, the average number of attended sessions per participant was high. The completion rate met our pre-specified target for determining feasibility. It should also be noted that most of the dropout occurred at the beginning of the intervention. Of the three non-completers, only one participated in more than one session and the reason for discontinuing the intervention was extra-therapeutic. Distinguishing between early and late dropouts could aid in understanding treatment attrition (Miller and Brennan, 2015). As suggested by Davis and Addis (1999), early dropouts may disagree with a treatment’s rationale, whereas late drop-outs may have problems with specific techniques. Motivation can also be a differentiating factor (Brennan et al., 2012). This question deserves consideration in future studies, in order to clarify potential similarities/differences in barriers to participation as experienced by both dropouts and completers.

### Study Limitations

This study should be interpreted in light of its limitations. First, this is an exploratory pilot study carried out with a small number of participants. Small samples may be appropriate for testing the feasibility of an intervention; however, they might overestimate treatment effects (Moore et al., 2011). Second, having no control group limits interpretation regarding the effect of the EF-CBT program on individuals. Third, the completion rate of the intervention was 70%. Despite attrition rates from 30 to 70% often being reported in studies of psychological interventions (Gustavson et al., 2012), we must be aware that attrition can bias the estimation of treatment effects and reduce generalizability (Leon et al., 2006). Four, the generalizability of this study is also limited by the inclusion of female participants only, most of them in middle age. We cannot exclude the possibility that male or adolescent response to this program may be different. Neither can conclusions regarding the impact of the intervention on patients without BED be drawn. By hypothesis, the EF-CBT program can promote emotional stability and the development of self-regulatory resources, such as inhibitory control, that can be useful for individuals with obesity in general. However, as this intervention was designed to address emotions related to eating behavior in the context of obesity, only further studies can clarify the effect of this approach on patients without overeating. Five, confounding variables, such as the presence/absence of previous psychological intervention and the current use of psychotropic medication, were not controlled. They could have impacted the report of alexithymia and emotional difficulties. Lastly, we did not examine whether participants still met diagnostic criteria for BED after the program. Considering that this approach was directed to individuals with BED, it would have been pertinent to determine whether there was a clinical change in this status.

These limitations, most of them due to the study being a pilot, should be addressed in a future randomized control trial, with some methodological improvements. Consideration should be given to the analysis of the role of comorbidities, such as depression and anxiety, on treatment outcomes. The integration of complementary measures to assess emotional processing, relying less on self-reporting, is another suggestion. It is known that individuals with low emotional awareness likely have difficulties completing self-report measures that require insight regarding their emotional status (Koch et al., 2015). Finally, we consider it pertinent to explore the features of the participants who benefit most from the EF-CBT intervention, as well as audit the clinical characteristics of the patients who dropped out of the group.

## Conclusion

We conclude that the EF-CBT program is feasible for use in obesity linked to BED to reduce impaired emotional functioning and relieve the dysfunction due to overeating. The positive long-term effect of the EF-CBT on outcome measures combined with meaningful participant feedback regarding their personal experiences with treatment, lead us to the conclusion that it would be worthwhile to proceed in research to establish the evidence-based needed to incorporate the EF-CBT in the treatment of obesity.

## Data Availability Statement

The datasets generated for this study are available on request to the corresponding author.

## Ethics Statement

The studies involving human participants were reviewed and approved by the Ethics Committee of Centro Hospitalar São João; reference number: CES 42-2011. The patients/participants provided their written informed consent to participate in this study.

## Author Contributions

ST and FV had full access to all of the data in the study and took responsibility for the integrity of the data and the accuracy of the data analysis. ST, CS, MG, and FV contributed to the study design. MP and MS contributed to the data collection. ST, CS, MG, MS, and FV contributed to the data analysis. ST, CS, and FV wrote the manuscript. All authors reviewed and approved the final version of the manuscript.

## Conflict of Interest

The authors declare that the research was conducted in the absence of any commercial or financial relationships that could be construed as a potential conflict of interest.
